# Supplementary material for: HLA3DB: comprehensive annotation of peptide/HLA complexes enables blind structure prediction of T cell epitopes
Source: Nat Commun. 2023 Oct 10;14:6349. doi: 10.1038/s41467-023-42163-z (PMC10564892; doi:10.1038/s41467-023-42163-z)
Supplement: Supplementary file 1 — Supplementary Information [file 41467_2023_42163_MOESM1_ESM.pdf]

**Supplementary Information**

# HLA3DB: comprehensive annotation of peptide/HLA complexes enables blind structure prediction of T cell epitopes

[†]Sagar Gupta[1,2], [†]Santrupti Nerli[1], Sreeja Kutti Kandy[1], Glenn L. Mersky[1], *Nikolaos G. Sgourakis[1,3]

[1]Center for Computational and Genomic Medicine, Department of Pathology and Laboratory Medicine, The Children's Hospital of Philadelphia, Philadelphia, PA, USA
[2]College of Arts and Sciences, University of Pennsylvania, Philadelphia, PA, USA
[3]Department of Biochemistry and Biophysics, Perelman School of Medicine, University of Pennsylvania, Philadelphia, PA, USA

[†]Contributed equally to this work.
***Correspondence**: Nikolaos G. Sgourakis
**Email:** nikolaos.sgourakis@pennmedicine.upenn.edu

**This PDF file includes:**

Supplemental Figure 1 to 12
Supplementary Table 1 and 2

## Supplementary Information

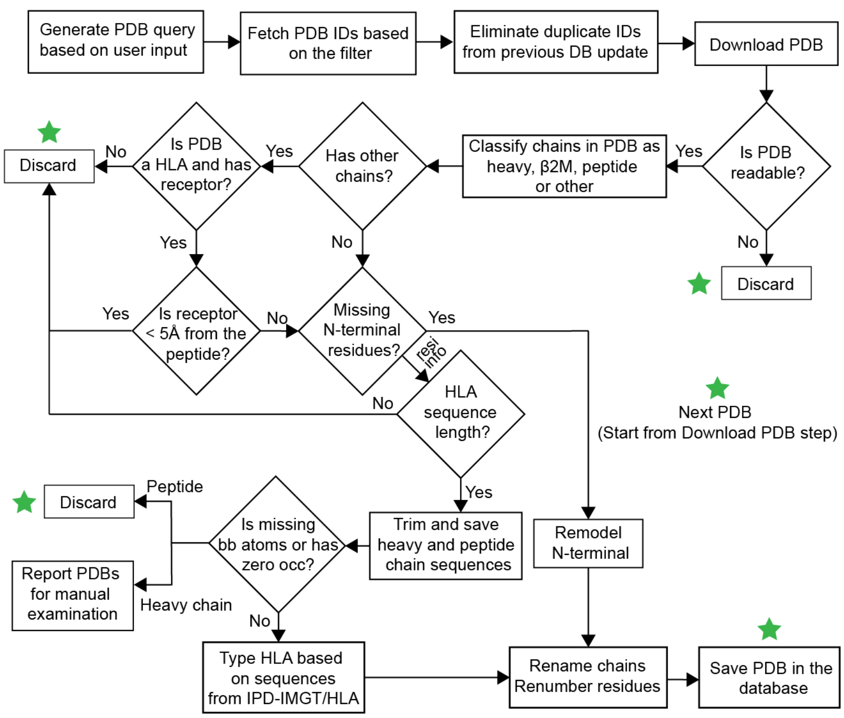


## Supplementary Figure 1

HLA3DB curation schematic implemented in Python using the RCSB PDB Search API[50].

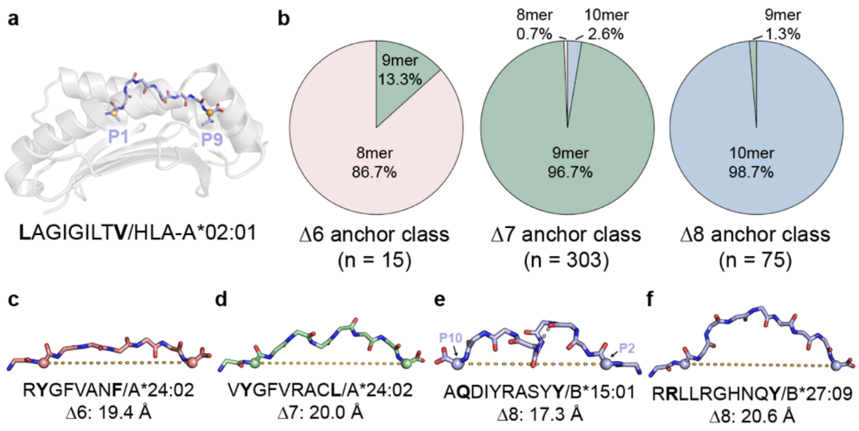


**Supplementary Figure 2**

Anchor class justification and special cases. **(a)** Non-canonical binding mode of the nonameric LAGIGILTV peptide bound to HLA-A*02:01 (PDB ID 2GTW). The MHC is colored in grey and shown as cartoon while the peptide backbone is colored in blue and shown as sticks. The Cα atoms of anchor positions (labeled) are shown as orange spheres. Anchor residues are further highlighted by bold text in the peptide sequence. **(b)** Pie charts showing the distribution of peptide lengths across the three anchor classes. **(c)** An extended conformation of a Δ6 octamer peptide (PDB ID 4F7T). **(d)** An extended conformation of a Δ7 nonamer peptide (PDB ID 2BCK). **(e)** A condensed conformation of a Δ8 decamer via an interpeptide $3_{10}$-helix (PDB ID 5VZ5). The peptide has been rotated 180°. **(f)** An extended conformation of a Δ8 decamer peptide (PDB ID 1JGD).

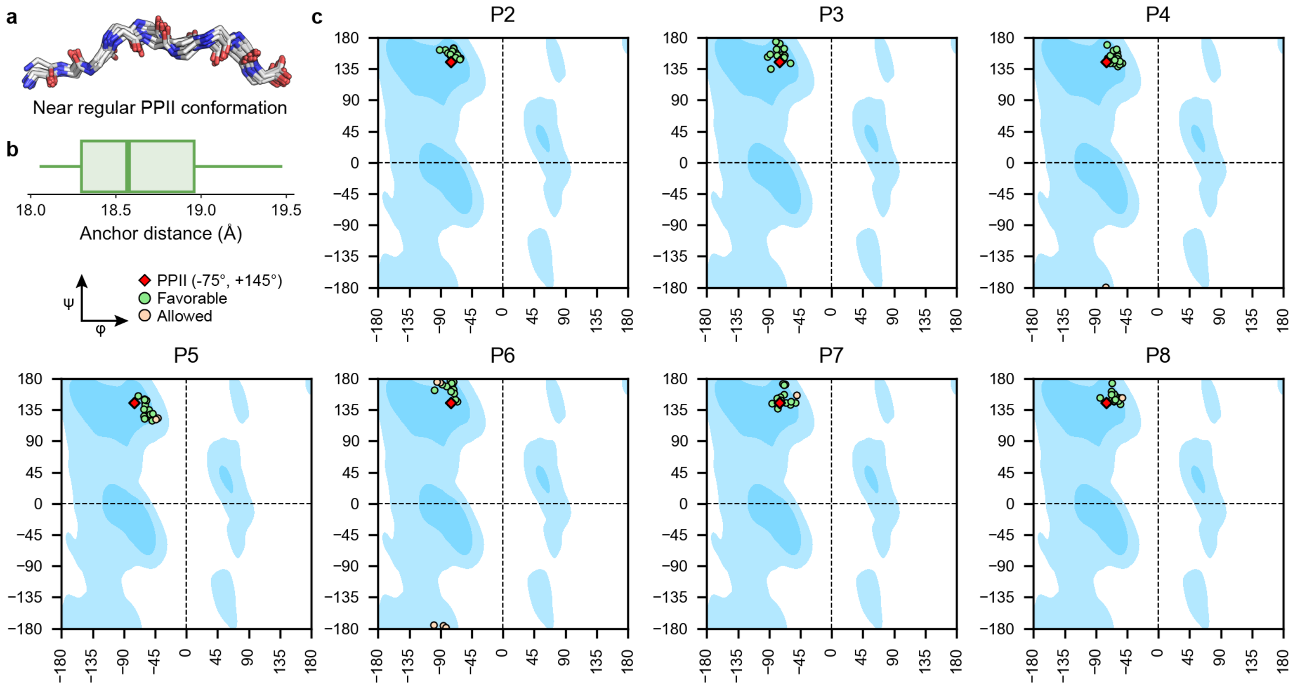


**Supplementary Figure 3**

Analysis of near regular PPII backbone conformations in Δ7 peptides. **(a)** Structural overlay of near regular PPII peptide backbones (n = 17). The MHC is not shown. **(b)** Anchor distance distribution of near regular PPII peptides with the center indicating the median. Whiskers extend to the furthest values that lie within the 75th and 25th percentile value ±1.5 times the interquartile range. **(c)** General Ramachandran plot showing dihedral angle pairs of the structures shown in (a). A legend is provided to the left of the plots.

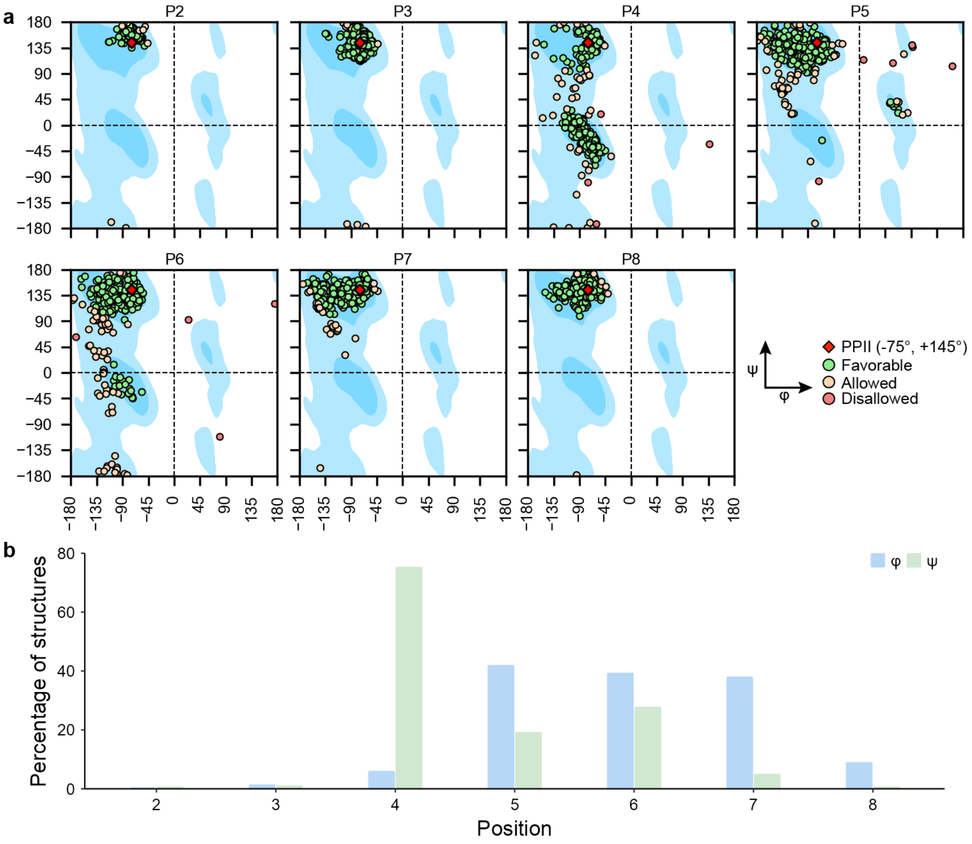


**Supplementary Figure 4**

Analysis of Δ7 peptides in HLA3DB. (**a**) General Ramachandran plots of all Δ7 peptides (n = 303). **(b)** Percentage of structures with a PPII deviation at a given dihedral angle and position.

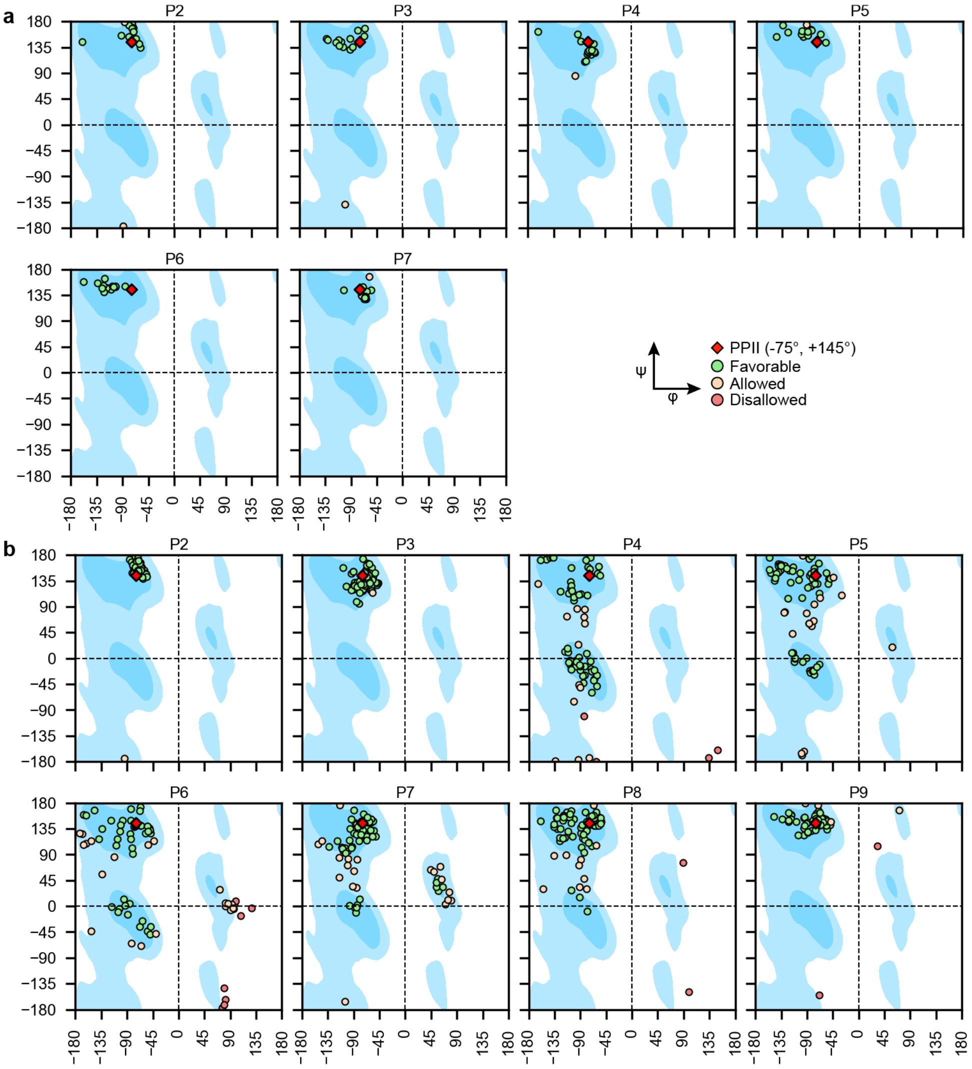


**Supplementary Figure 5**

Analysis of Δ6 and Δ8 peptides in HLA3DB. **(a)** General Ramachandran plots of all Δ6 peptides (n = 15). **(b)** General Ramachandran plots of all Δ8 peptides (n = 75). Plots are colored and formatted identically to (a).

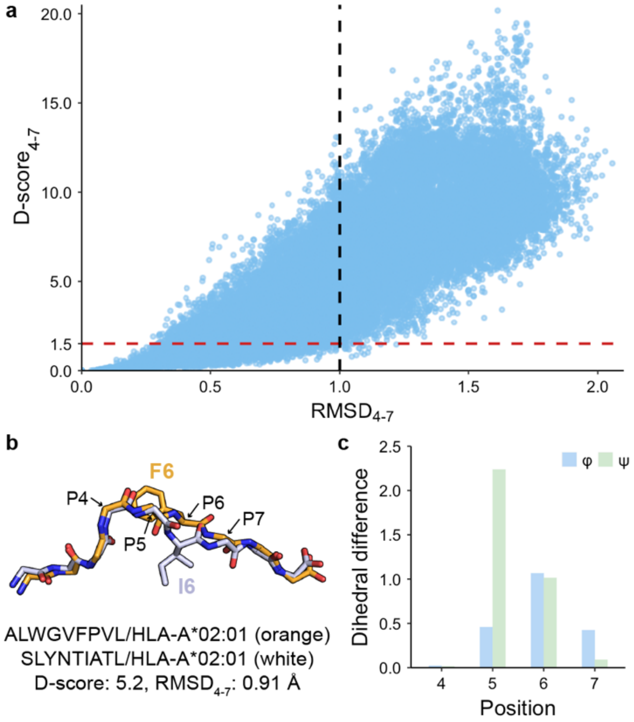


**Supplementary Figure 6**

Comparison of D-score and RMSD. **(a)** Pairwise comparison of D-score and backbone heavy atom RMSD for P4 to P7. **(b)** Exemplar superposition of ALWGVFPVL/HLA-A*02:01 (PDB ID 1I7T, orange) and SLYNTIATL/HLA-A*02:01 (PDB ID 5NMH, white). The MHC is not shown. **(c)** Dihedral difference by position and angle for the exemplar structural superposition shown in (b).

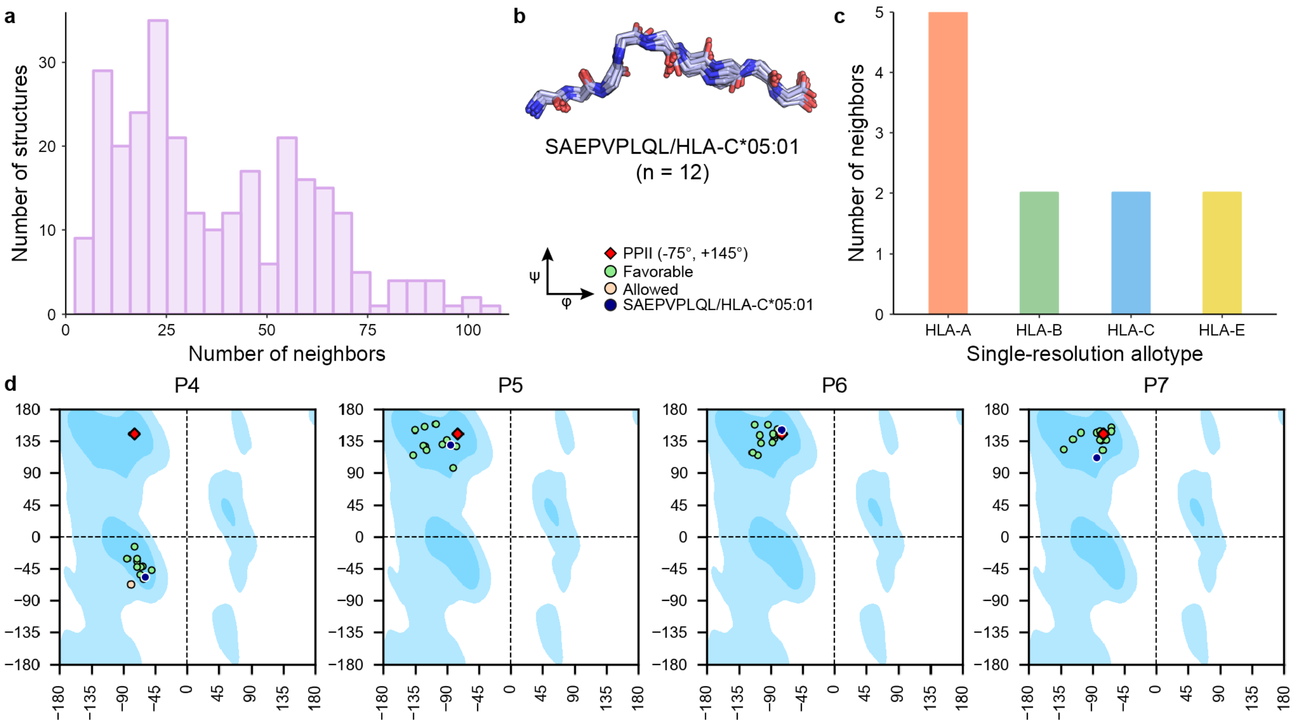


**Supplementary Figure 7**

Structural conservation of peptide backbones occurs across allotypes. **(a)** Number of neighbors for every Δ7 peptide. **(b)** Structural superposition of neighboring backbones to the exemplar peptide (PDB ID 5VGD) via peptide backbone heavy atoms. The MHC is not shown. **(c)** Number of neighbors of the exemplar structure by single-resolution allotype. **(d)** Ramachandran plots of P4 to P7 of the neighbors (green/cream) and exemplar structure (dark blue).

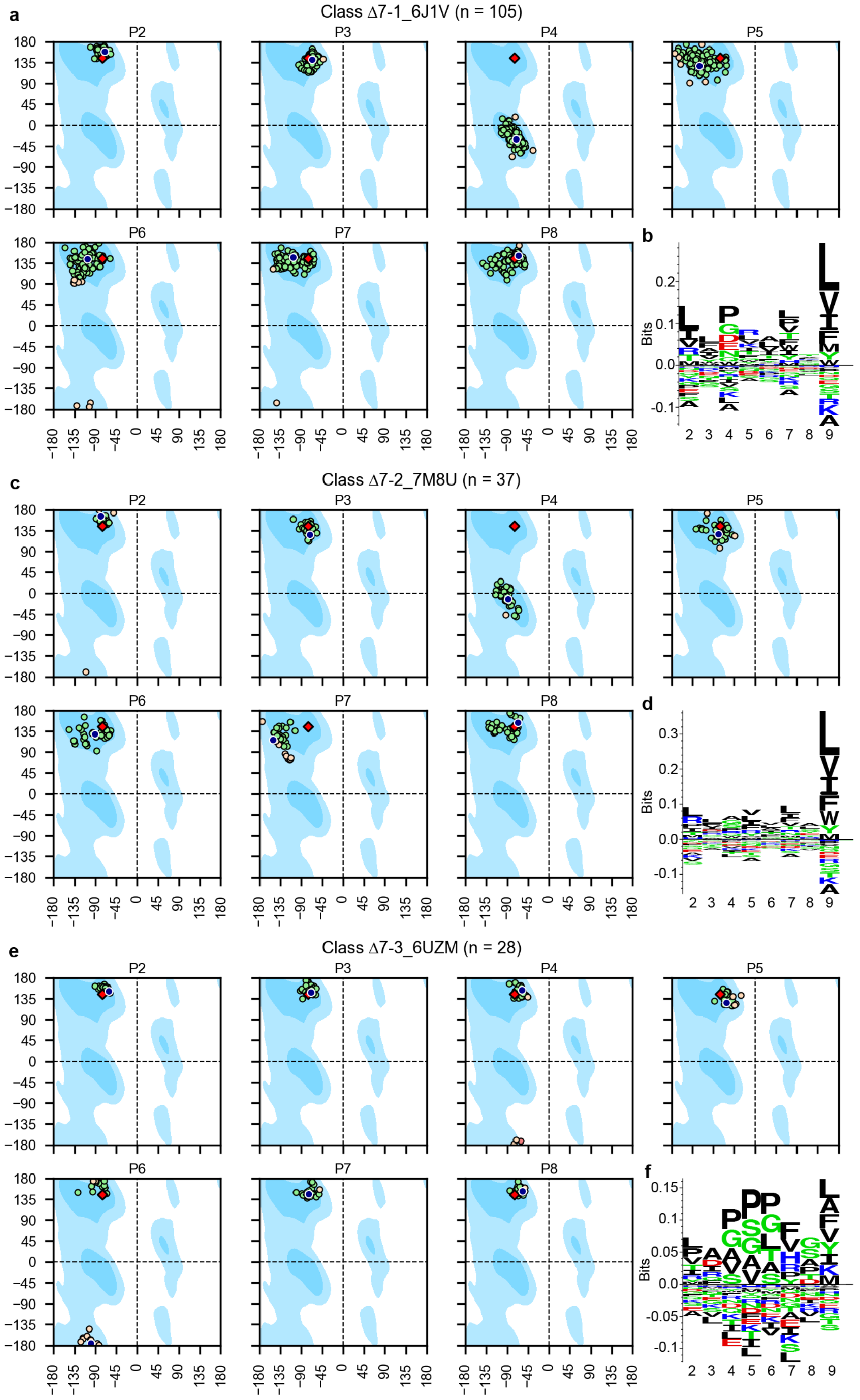


**Supplementary Figure 8**

Additional information of the three most common backbone classes among the Δ7 peptides. **(a)** Ramachandran plot of the most common backbone class (n = 105). **(b)** Sequence logo of peptides shown in

(a). **(c)** Ramachandran plot of the second most common backbone class (n = 37). **(d)** Sequence logo of peptides shown in (c). **(e)** Ramachandran plot of the third most common backbone class (n = 28). **(f)** Sequence logo of peptides shown in (e). All sequence logos were created using Seq2Logo[89].

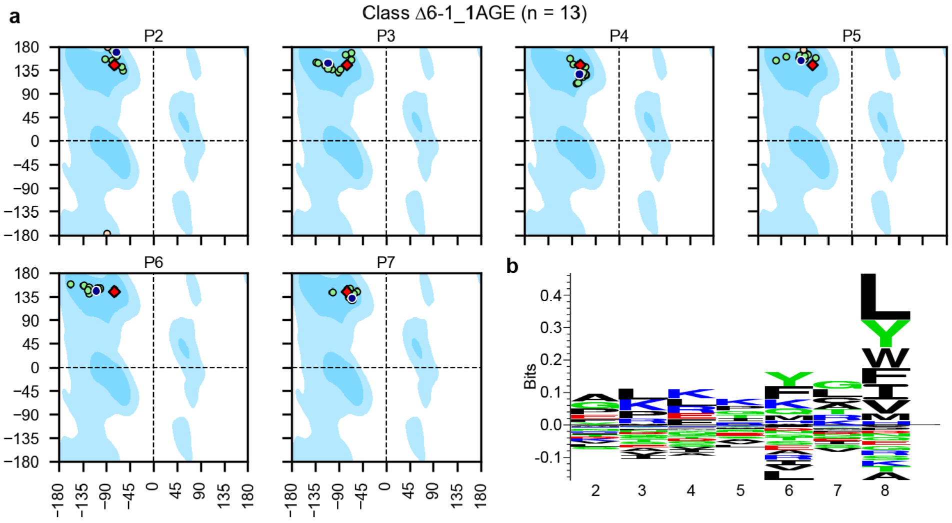


**Supplementary Figure 9**

Additional information of the most common backbone classes among the Δ6 peptides. **(a)** Ramachandran plot of the most common backbone class (n = 13). **(b)** Sequence logo of peptides shown in (a). Created using Seq2Logo[89].

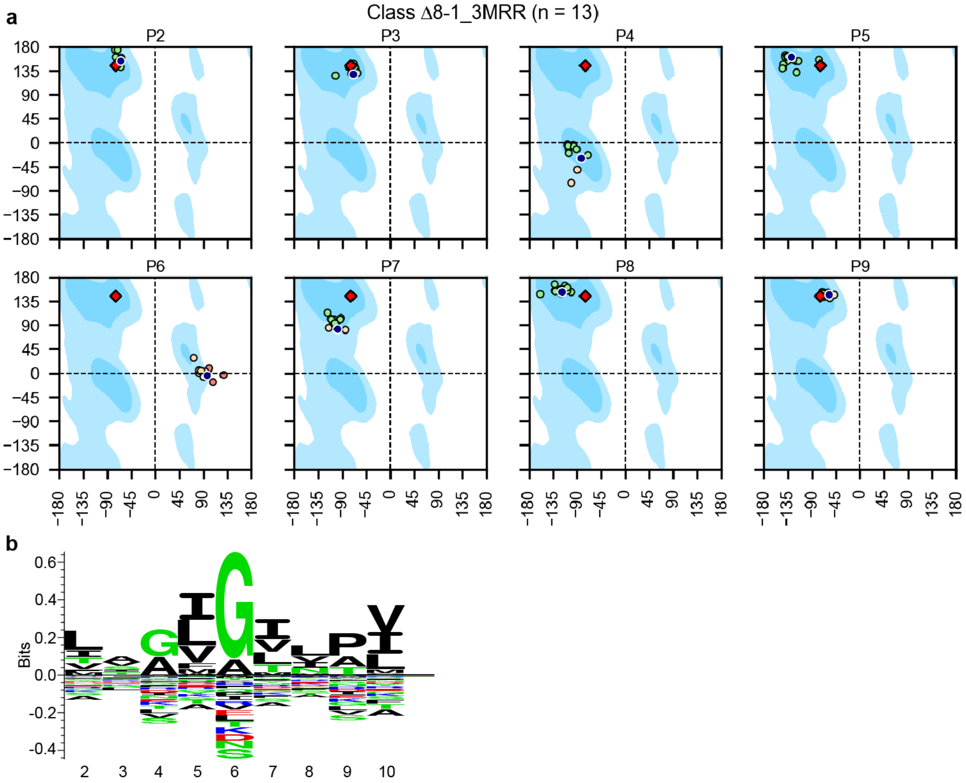


**Supplementary Figure 10**

Additional information of the most common backbone classes among the Δ8 peptides. **(a)** Ramachandran plot of the most common backbone class (n = 13). **(b)** Sequence logo of peptides shown in (a). Created using Seq2Logo[89].

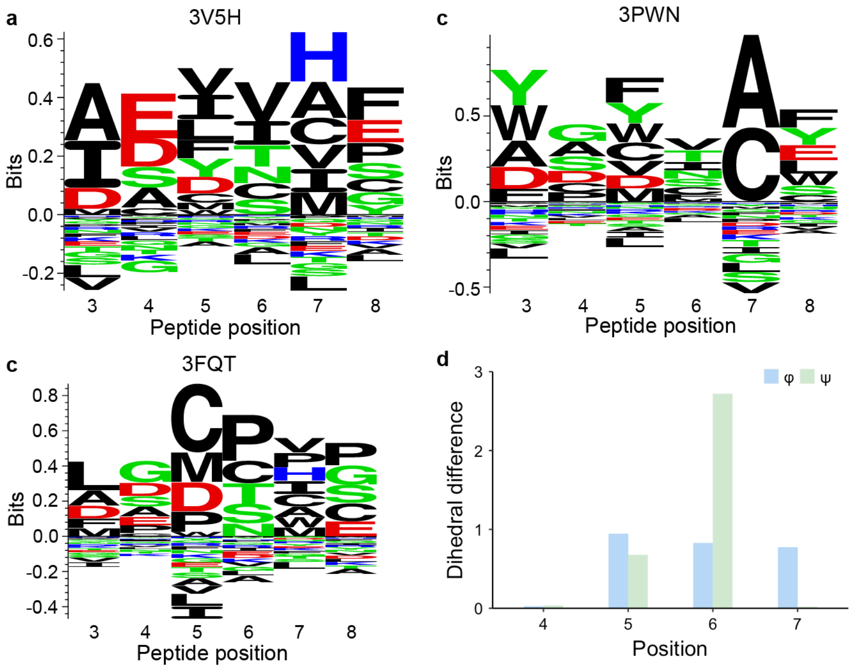


**Supplementary Figure 11**

Additional analysis of the exhaustive structural modeling results. Peptide sequence logos from structural modeling results (n = 7,840) of **(a)** 3V5H, **(b)** 3PWN, and **(c)** 3FQT. Created using Seq2Logo[89]. **(d)** Dihedral difference by position and angle for 5HHQ and 6VR1. A superposition of these discrete peptide backbones are shown in Fig. 3d.

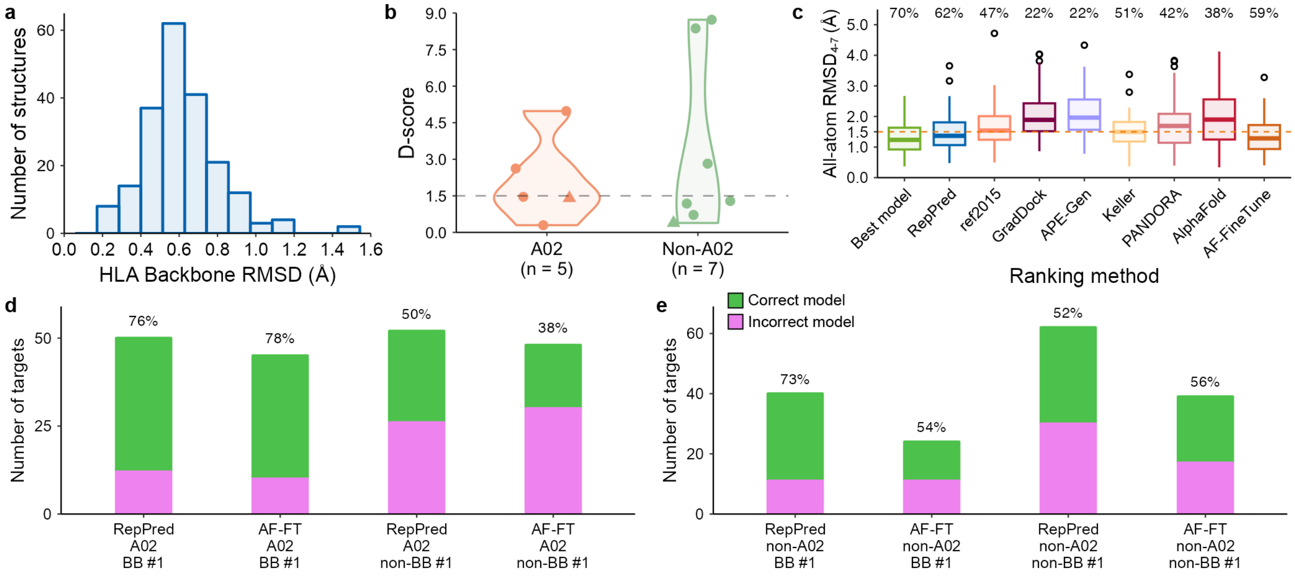


**Supplementary Figure 12**

Additional analysis of the accuracy of RepPred and its comparison to AF-FT. (**a**) Distribution of HLA backbone heavy atom RMSD between the RepPred model and the corresponding target crystal structure for all targets. **(b)** Overall results of blind testing of RepPred. Points depicted as triangles are elaborated in Fig. 4c and 4e. (**c**) Boxplots showing the distribution of peptide all-heavy-atom RMSD of RepPred and six state-of-the-art methods, sorted by publication date, for A02 targets with the center indicating the median (Best model, Random, RepPred, ref2015: n = 102; GradDock: n = 90; APE-Gen: n = 99; Keller: n = 101; PANDORA: n = 100; AlphaFold: n = 101; AF-FineTune: n = 93). Whiskers extend to the furthest values that lie within the 75th and 25th percentile value ±1.5 times the interquartile range and outliers are shown in black circles. An orange dashed line is at an RMSD of 1.5 and the percentage of models under this threshold for each method is listed above the respective boxplot. Comparison of RepPred and AF-FT for the most common backbone conformation (PDB ID 6J1V) and all other backbones for **(d)** A02 targets and **(e)** non-A02 targets.

| Class | # of Structures | Peptide Conformation | Median D-score |
|---|---|---|---|
| Δ6-1_1AGE | 13 | PBB,PPB | 0.14 |
| Δ6-2_4F7T | 1 | BBB | 0 |
| Δ6-3_1E28 | 1 | PPP | 0 |
| | | | |
| Δ7-1_6J1V | 105 | ABBB,ABBP,APBB | 1.03 |
| Δ7-2_7M8U | 37 | APBB,APPB,DPPB | 0.92 |
| Δ7-3_6UZM | 28 | PPBP,PPPP | 0.43 |
| Δ7-4_1UXS | 27 | PBAP,PBDP,PPAB,PPDB,PPDP | 1.02 |
| Δ7-5_5IEK | 19 | ABPB,ABPP,ADPB,DBPB,DDPP | 0.64 |
| | | | |
| Δ8-1_3MRR | 13 | ABLBB,ABLPB,DBLBB,DBLPB | 0.65 |
| Δ8-2_5FDW | 8 | APPPP | 0.68 |
| Δ8-3_5C0I | 6 | BABPP | 0.73 |
| Δ8-4_3DX7 | 5 | BBPLB,BBPLP,BPPLP,PPPLP | 1.09 |
| Δ8-5_3OXR | 4 | PPAPP,PPDPP | 0.32 |

**Supplementary Table 1**

Peptide conformations are included if they are present in at least 10% of structures in a given class and labeled according to previously established regions of the Ramachandran plot[54]. The median D-score was calculating by taking median of the D-score between each member of the class and the corresponding discrete peptide backbone.

| Peptide position | Amino acids |
|---|---|
| **3** | A, D, F, I, L, M, P, W, Y |
| **4** | A, C, D, E, G, P, S, W |
| **5** | C, D, F, I, L, M, P, V, W, Y |
| **6** | C, F, I, L, M, N, P, S, T, V, W |
| **7** | A, C, F, H, I, L, M, P, V, W, Y |
| **8** | C, E, F, G, L, P, S, W, Y |

**Supplementary Table 2**

Amino acids used for exhaustive structural modeling of likely peptide sequence combinations in the HLA-A*02:01 groove derived from an amino acid similarity matrix for peptide/MHC binding[58].
